# Long-Term Observation and Treatment of Epithelioid Haemangioendothelioma of the Mediastinum: A Case Report

**DOI:** 10.3389/fsurg.2021.678572

**Published:** 2021-10-05

**Authors:** Qiuli Zhi, Zhoupeng Ma, Guansheng Lin, Jiangfeng Pan, Bingye Chen

**Affiliations:** ^1^Department of Radiology, Affiliated Cixi Hospital, Wenzhou Medical University, Cixi, China; ^2^Department of Radiology, Jinshan TCM-Integrated Hospital of Shanghai City, Shanghai, China; ^3^Department of Radiology, Jinhua Hospital, Zhejiang University School of Medicine, Jinhua, China; ^4^Department of Surgery, Jinshan TCM-Integrated Hospital of Shanghai City, Shanghai, China

**Keywords:** epithelioid hemangioendothelioma, mediastinum, diagnosis, treatment, case report

## Abstract

Epithelioid haemangioendothelioma is a rare angiogenic tumour originating from vascular endothelial or pre-endothelial cells, and it can occur anywhere in the body, such as the liver, lung, bone, spleen, lymph nodes, parotid gland, and thyroid. In the fifth revision of the WHO classification, epithelioid haemangioendothelioma (EHE) was described as a malignant vascular neoplasm composed of epithelioid endothelial cells, distinct from epithelioid angiosarcoma. We, herein, report one patient with EHE of the left upper mediastinum who underwent resection and radiotherapy during the first therapeutic process. Multiple metastases occurred in the thoracic vertebrae 6 years later, and resection and multiple radiotherapies were performed. The condition of the patient remained stable at the last review in October 2020, and it has been more than 8 years since her first admission. The reasonable “take-away” lessons from the case are active treatment and prolonged surveillance.

## Introduction

Epithelioid haemangioendothelioma is a rare angiogenic tumour originating from vascular endothelial or pre-endothelial cells (1, 2), with an incidence of <1 in 1 million individuals per year (2). In 1982, Weiss and Enzinger described epithelioid haemangioendothelioma (EHE) as a vascular tumour of the bone and soft tissue showing features between haemangioma and angiosarcoma (3, 4). However, EHE was described as a malignant vascular tumour distinct from epithelioid angiosarcoma in the fifth revision of the WHO classification (5). The primary tumour can occur anywhere in the body, and it arises in the lung, bone, and liver in more than 65% of all cases (1, 6), while the mediastinum is a rather rare site (7, 8). Due to its rarity, atypical symptoms, and non-specific laboratory indicators, preoperative misdiagnosis is usual.

The aim of the present study was to report a case of mediastinal EHE that is characterised by long-term observation and treatment.

## Case Presentation

**Figure 1:**
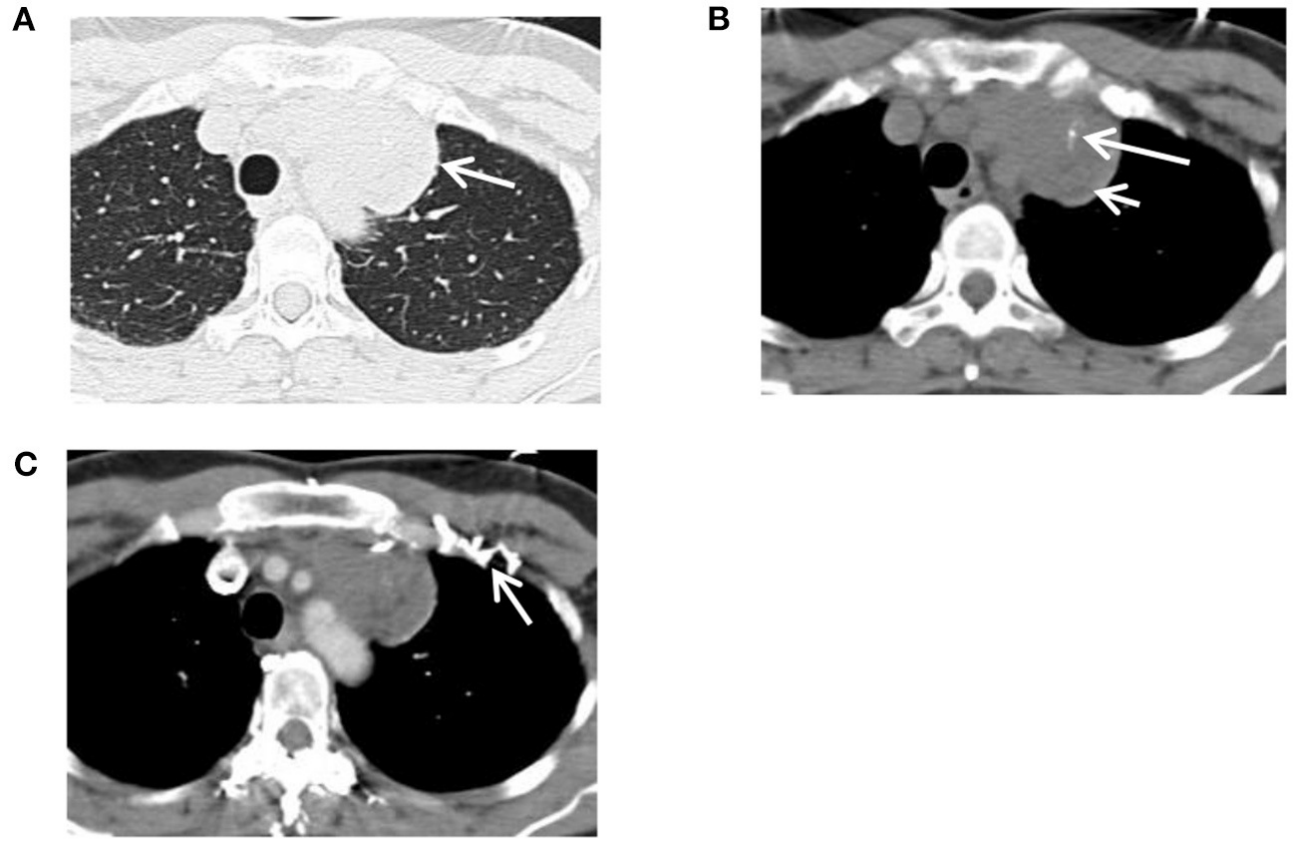
CT of a 49-year-old female patient with mediastinal epithelioid haemangioendothelioma (EHE) at the first treatment. **(A)** An unenhanced CT revealed a mass in the left anterior mediastinum, and the interface between the lung and the mass was smooth (arrow). **(B)** Spotted calcification (long arrow) and fatty foci (short arrow) were observed inside the mass, and the lesion was closely connected with mediastinal vessels and the anterior chest wall. **(C)** An enhanced CT of the venous phase indicated a mass with asymmetrical mild enhancement, and the collateral circulation of the left upper chest wall was opened abnormally (arrow).

A 49-year-old previously healthy Chinese woman was admitted to the hospital on July 28, 2012, due to 3 months of mild, dull pain and discomfort in the left upper chest. She did not have any relevant family history. On admission, a physical examination showed mild swelling of the left anterior upper chest wall and no other abnormalities. Routine blood, urine, stool, and haepatic and renal function tests revealed normal results. The serum levels of tumour markers, including CA125, CEA, AFP, CA19-9, CA242, CA211, NSE, SCC, CA15-3, and HCG, were all within normal ranges. A contrast-enhanced chest CT revealed one solid spherical mass (59 × 55 × 66 mm) with spotted calcification and fatty foci in the left anterior mediastinum, which showed asymmetrical mild enhancement. The lesion was closely connected with mediastinal vessels and the anterior chest wall, the left innominate vein was compressed locally, and the collateral circulation of the left upper chest wall was engorged. Enlarged lymph nodes of the mediastinum or evident abnormal density shadows of the thymus area could not be observed, and other sites, including the thoracic vertebrae, showed no obvious abnormalities (Figures 1A–C). The CT diagnosis was “teratoma.” No other subsequent imaging examination was performed. Considering the risk of puncture pathology due to the proximity of the lesion to the large vessels and the diagnostic uncertainty of its accuracy, preoperative puncture pathology was not performed.

The patient and her family were informed of her condition. According to the current diagnosis and the general situation of the patient, she required surgical treatment to remove the tumour and make a pathological diagnosis. The patient and her family agreed and expressed willingness to accept the risk of surgery. Therefore, an anterior mediastinal tumour resection was performed through the anterior chest wall under general anaesthesia on August 5, 2012. After successful anaesthesia, the patient took a supine position. A median longitudinal incision of the anterior chest wall of ~19 cm was made, the sternum was sawn longitudinally, and then the surgical incision was enlarged bluntly to display the lesion fully. A tumour of ~60 × 56 × 57 mm was found in the left anterior mediastinum, extending into the space between the left common carotid artery and the left subclavian artery, and adhering closely to the blood vessels of the mediastinum and anterior chest wall. The left innominate vein was compressed evidently, wrapped locally, and thinned, but evident invasion or destruction cannot be observed. Proliferative small blood vessels can be observed around the mass. The tumour was dissociated carefully, and all small bleeding sites were fully ligated for haemostasis. The tumour was completely separated and removed eventually, and regional lymphadenectomy was performed at the same time.

**Figure 2:**
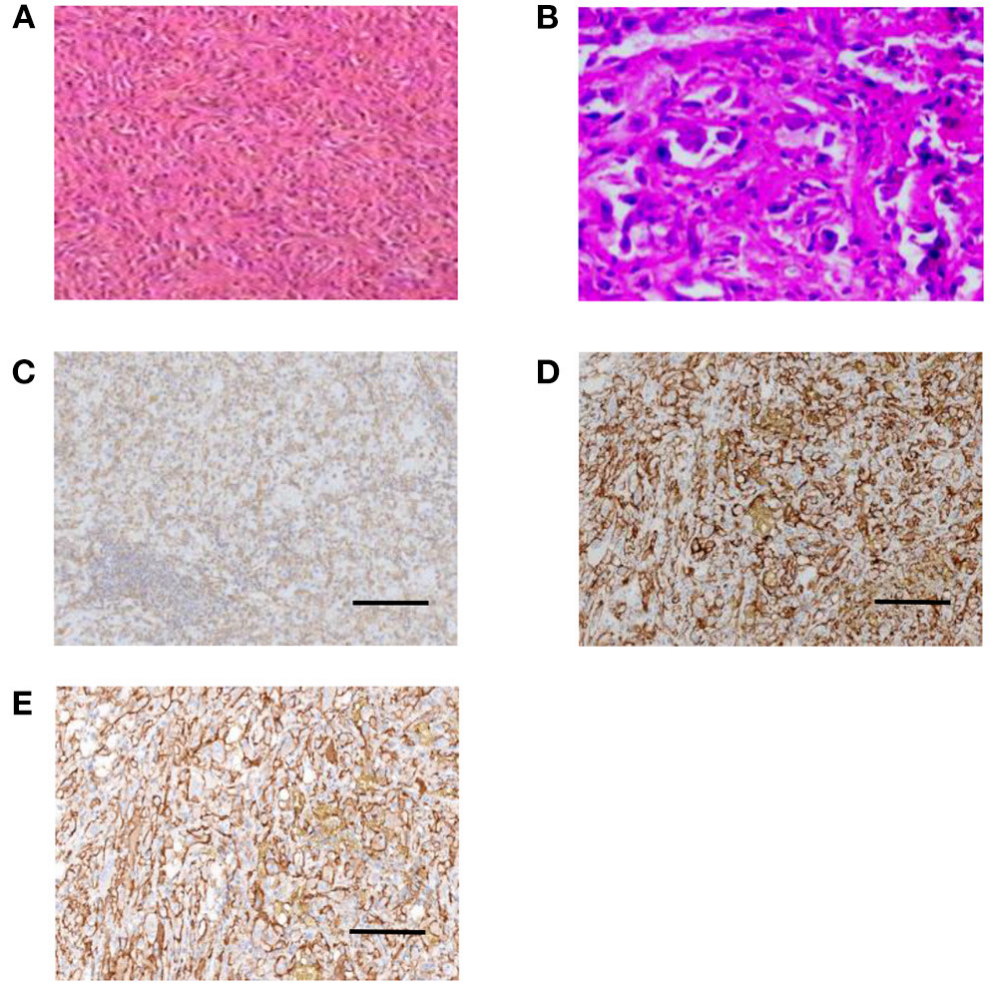
Pathology of a 49-year-old female patient with mediastinal EHE at the first treatment. **(A)** Histology showed that the tumour was composed of epithelioid cells and fusiform cells (HE, × 200). **(B)** Small focal necrosis and tumour thrombi in the vessels and nuclear division were observed (HE, × 400). **(C–E)** Immunohistochemistry showing tumour cells positive for CD34 **(C)**, CD31 **(D)**, and vimentin **(E)**, scale bar = 200 μm.

Postoperative pathology showed that the lesion was well-demarcated. Histology showed that it was composed of epithelioid cells and fusiform cells, with small focal necrosis and tumour thrombi in the vessels. Nuclear division was observed, with an average of less than 1/10 high power field (HPF) (Figures 2A,B). Postoperative pathology confirmed that radical resection (R0) was achieved. The immunohistochemical results were as follows: vimentin (++) (Figure 2C); CD34 (++) (Figure 2D); CD31 (++) (Figure 2E); F8 (+); cytokeratin (AE1/AE3) (CK (AE1/AE3)) (–); epithelioma antigen (EMA) (–); smooth muscle actin (SMA) spindle cells (+); desmin (–); calretinin (–); CD5 (–); and S-100 (focal+). The Ki67 index was ~20% (+). The final pathological diagnosis was mediastinal EHE.

The patient and her family were informed of the pathological diagnosis and the subsequent therapeutic schedule, and the possible effects and side effects of adjuvant radiotherapy and adjuvant chemotherapy were explained in detail. They agreed to accept adjuvant radiotherapy but refused chemotherapy, so intensity-modulated radiation therapy (IMRT) (6 MV-X-ray DT40 Gy/10 F) was conducted from September 3, 2012, to December 16, 2012 and the radiotherapy equipment was Varians' Clinac 21EX accelerator (Varian Medical Systems, USA). The patient had an uneventful recovery, and her left upper chest pain and discomfort disappeared. Upon discharge, the patient and her family were informed that she should be followed up every 3 months in the first 2 years after the operation for a physical examination, chest CT, and tumour marker detection. Subsequently, she should be reviewed every 6 months. She did not accept any other treatment, and regular reviews on May 6, 2014 and March 4, 2016 showed no definite metastasis or recurrence by unenhanced CT scans of the chest. The serum levels of tumour markers, including carbohydrate antigen 125 (CA125), carcinoembryonic antigen (CEA), alpha fetoprotein (AFP), carbohydrate antigen19-9 (CA19-9), carbohydrate antigen242 (CA242), carbohydrate antigen211 (CA211), neurogen specific enolase (NSE), squamous cell carcinoma (SCC), carbohydrate antigen15-3 (CA15-3), and human chorionic gonadotropin (HCG), were all within normal ranges. The patient and her family refused 18F-fluorodeoxyglucose (FDG) PET/CT because of the concerns about economic cost and radiation injury.

**Figure 3:**
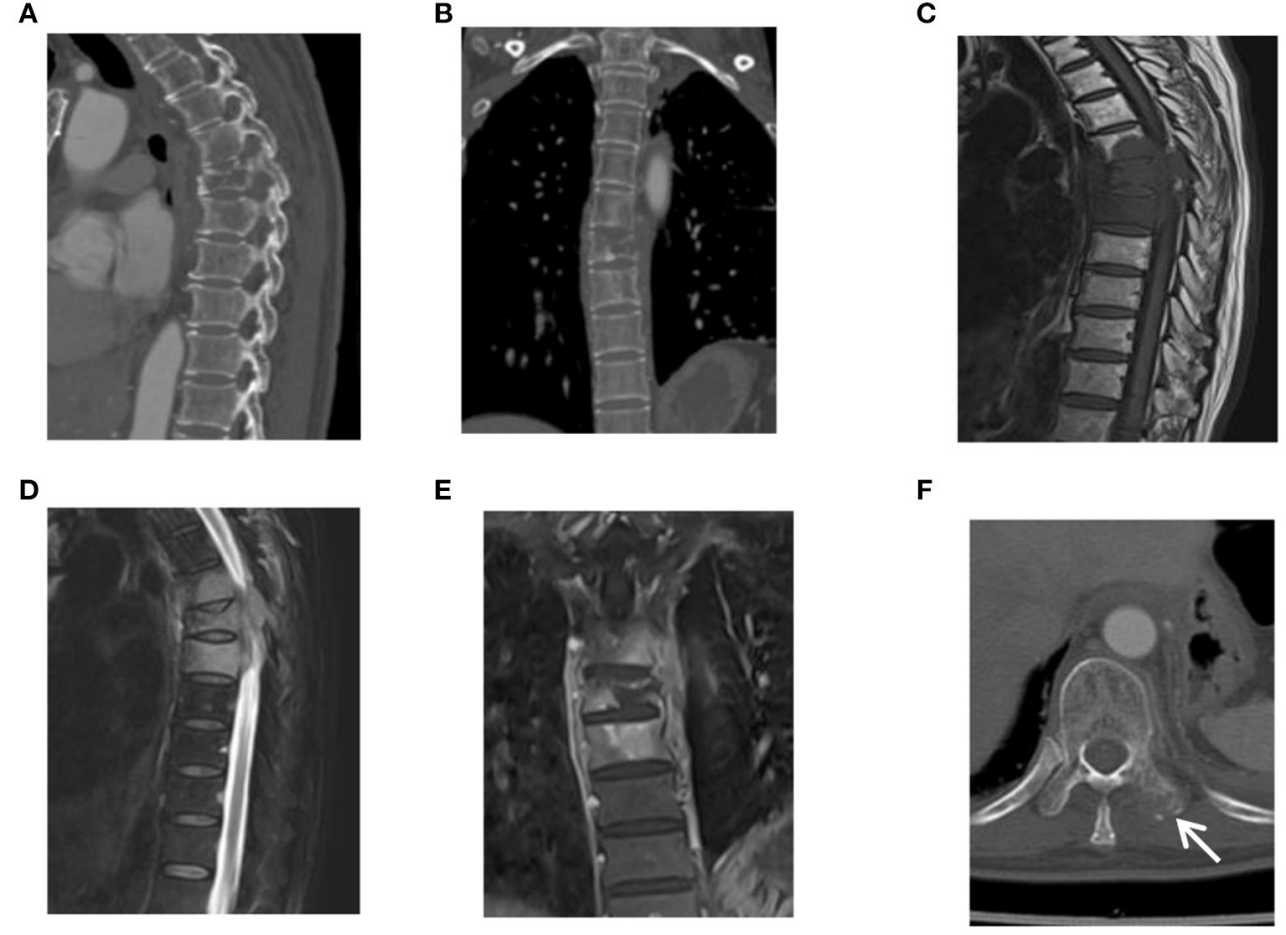
CT and MRI of a 49-year-old female patient with mediastinal EHE before the second treatment. **(A–D)** CT **(A,B)**, and MRI **(C,D)** revealed multifocal lesions of the thoracic vertebrae 5–7. **(E)** An enhanced MRI of the venous phase revealed multifocal lesions of the thoracic vertebrae with obvious asymmetrical enhancement. **(F)** The CT indicated a local ruin of the left transverse process of the 10th thoracic vertebra (arrow).

**Figure 4:**
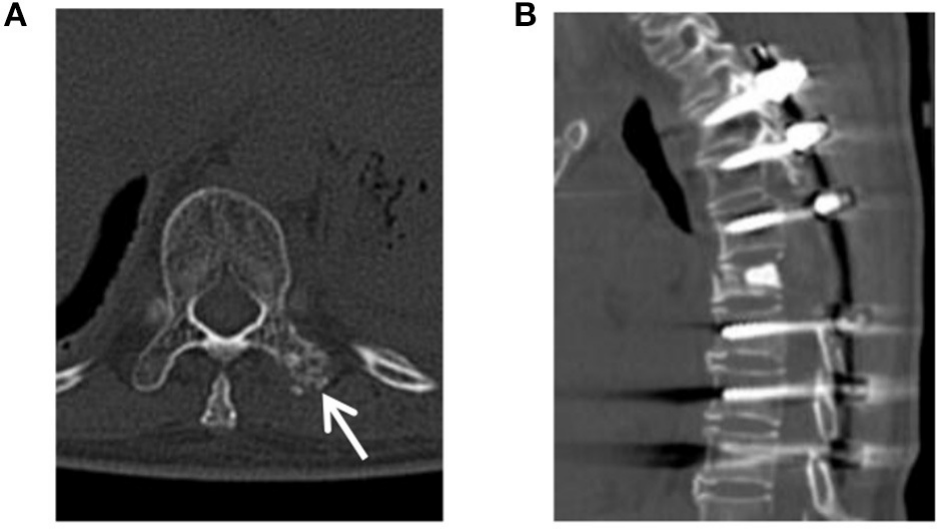
CT of a 49-year-old female patient with mediastinal epithelioid haemangioendothelioma (EHE) at the latest review. **(A)** CT revealed that the lesion of the left transverse process of the T10 vertebra *was* small and localised (arrow). **(B)** A CT image of a sagittal reconstruction showed that the internal fixations of thoracic vertebrae were stable.

**Figure 5:**
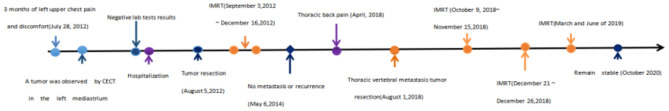
The schedule of diagnosis and treatment of a 49-year-old female patient with mediastinal EHE.

Thoracic back pain and discomfort occurred in April 2018 and became increasingly evident 6 years after the first operation. The serum levels of tumour markers were normal, except for CA125, which increased obviously (87.9 U/ml, normal < 35 U/ml) on July 15, 2018, and all other laboratory examinations were normal. The CT and MRI subsequently revealed multifocal lesions of the T5–7 vertebrae (Figures 3A–E) and the left transverse process of the T10 vertebra (Figure 3F). The patient and her family refused an 18F-FDG PET/CT examination as before; the main reason may still be the concerns about costs and radiation. Then, they were informed that thoracic spinal surgery and pathological diagnosis were needed to determine whether metastases had occurred. They all agreed. Therefore, posterior resection, decompression, and internal fixation for thoracic vertebral malignant tumours of the T5–7 vertebrae were conducted on August 1, 2018. The pathological diagnosis was metastatic EHE. The patient and her family refused to accept chemotherapy as before and agreed to radiotherapy. Intensity-modulated radiation therapy for the thoracic vertebral tumour area (6 MV-X-ray DT40 Gy/20 F) was conducted from October 9, 2018 to November 15, 2018. The radiotherapy process of the patient was smooth, and the level of CA125 on November 22, 2018, decreased obviously compared with that on July 15, 2018 (51.1 U/ml and 87.9 U/ml, normal < 35 U/ml). After the IMRT course from December 21, 2018 to December 26, 2018, the level of CA125 continued to decrease to 48.3 U/ml on December 29, 2018, and all other laboratory examinations were normal. Since the lesion of the left transverse process of the T10 vertebra was small and localised, surgical treatment was not performed, and the patient underwent two rounds of IMRT in March and June 2019. Until the last review on October 22, 2020, the condition of the patient was stable. In addition to occasional chest and back dull pain, discomfort, and an inability to bear weight, other complications of spinal surgery, such as spinal cord injury and limb sensory and motor dysfunction, did not occur. Regarding the side effects of radiotherapy, except for mild radiation pneumonia and radiation pleurisy, other major side effects did not occur, including myelosuppression, skin injury, and spinal cord injury. The CT showed that the lesion of the left transverse process of the T10 vertebra remained stable (Figures 4A,B), and there was no recurrence or other metastasis. For the residual metastatic focus in the transverse process of the T10 vertebra, the patient and her family were told to observe closely and cheque over time if abnormal symptoms appeared. It has been more than 8 years since the first admission of the patient. The schedule of diagnosis and treatment is shown in Figure 5 below.

## Discussion

Epithelioid haemangioendothelioma was initially believed to be an aggressive form of bronchoalveolar cell carcinoma invading adjacent blood vessels and small airways, hence the name intravascular bronchioloalveolar tumour (9, 10). However, the development of pathology proved that EHE could occur anywhere in the body, such as the liver, lungs, skin, bone, spleen, pleura, lymph nodes, thyroid, parotid gland, and cerebellum (11–13). In the 2020 WHO classification of sarcomas, EHE is distinguished from other vascular tumours and defined as an independent disease (5). Furthermore, EHE represents <1% of all vascular tumours and generally manifests as multiple lesions, such as tumours originating from the liver, lungs, and bone, while a single lesion is relatively rare, especially solitary lesions originating from the mediastinum (7, 8).

The aetiology of EHE remains unclear. Different angiogenic stimulators and monocyte chemoattractant protein-1 might stimulate the angiogenesis of endothelial cells and lead to the occurrence of lesions (14, 15). Some studies have suggested that abnormal cloning and gene mutation play important roles in tumour development, but they have not yet been recognised (1). Moreover, radiation, defunctionalised arteriovenous fistula, foreign bodies, carotid endarterectomy, and intravascular prosthesis have been suggested to be predisposing factors (16). What is confusing is that the present case lacked any of the above factors, and the cause of the disease of the patient is unknown. Histologically, EHE is characterised by cords and nests of spherical to slightly spindled epithelioid cells embedded in a myxoid matrix (7). Immunohistochemically, the endothelial marker CD31 shows good sensitivity and specificity for identifying vascular neoplasms, including EHE. Some cases of EHE stain negatively for CD31 but are positive for other vascular markers, such as CD34 and factor VIII-related antigen (17).

The clinical symptoms of mediastinal EHE are non-specific, such as chest pain, coughing, dyspnoea, and hoarseness of the voice, mainly due to the local oppression or invasion of adjacent structures (8). Low-grade fever and generalised weakness occur occasionally, but night sweats are rare, and some asymptomatic patients are diagnosed incidentally in imaging investigations performed for other reasons (7). If metastasis occurs, the corresponding symptoms will appear, such as pain, mass, haemorrhage, and other abnormalities (1, 2, 8). In the present case, the clinical symptoms of the early stage were mild and atypical and only showed mild dull pain and discomfort in the left upper chest, but obvious chest back pain occurred after thoracic vertebra metastasis in the late stage.

The preoperative misdiagnosis of EHE is usually due to its low incidence, lack of specific oncological markers, and unclear pathogenesis (1, 13). However, the improvement of medical imaging equipment and diagnostic technology has made it possible for medical imaging, including CT, MRI, and F-FDG-PET to show certain pathological characteristics of EHE (1, 6–8). Mediastinal EHE is typically expressed as a soft-tissue mass with a well-defined margin on CT and MRI, and it is less likely to invade the surrounding tissues due to its relatively indolent clinical course compared to EHE originating from liver, lung, and soft tissue (7, 8, 18). Moreover, spotted calcification and fatty foci can be detected in some cases, while pleural effusion or enlarged lymph nodes of the mediastinum are rare (19); thus, EHEs are easily misdiagnosed as benign tumours. In the present case, the false diagnosis of a “teratoma” was made as spotted calcification, and fatty foci were observed on CT. On enhanced CT and MRI, EHE can demonstrate varied enhanced modes, including isoenhancement, progressively intense enhancement, ring-like enhancement, and even non-contrast enhancement (7, 8, 18, 19), depending on the composition and distribution of different tissues inside the focus. Increased uptake of 18F-FDG in the tumour indicates the nature of the malignancy (18, 20).

The prognosis of EHE differs greatly and is related to the extent of tumour cell differentiation, the site and extent of tumour involvement, metastasis, and specific individual factors (1, 2, 7, 8). In general, the biologic behaviour of EHE is typically a more indolent cancer resulting in a mean survival of 4.6 years, ranging from 6 months to 24 years (1). Metastases and unresectability are associated with poor prognosis, and extensive metastases, haemorrhage, and respiratory failure are the main causes of death (19, 21, 22).

Since EHE is a rare malignancy that can occur in any organ system, no optimal treatment strategy has been established (2). Surgery is the first choice of treatment, and radiation therapy after surgical resection is chosen for localised EHE to control the residual disease given the recurrence of EHE (1, 8, 23). Chemotherapy is preferred in cases of widespread disease (1, 19, 21). In the present case, radiation therapy was conducted after the surgical resection of the primary lesion, and the patient remained stable for 6 years. While the multifocal metastasis of the thoracic vertebrae occurred, the patient underwent another surgical resection and multiple adjuvant radiotherapies. She and her family refused to accept adjuvant chemotherapy, and the main reason might have been uncertainty of the curative effects and concern about economic costs. In this case, the therapeutic effect was satisfactory; her condition was stable up to her last review on October 22, 2020; the serious side effects of radiotherapy, including spinal cord injury, nerve injury, and myelosuppression, did not occur.

The limitations of this report are as follows: There was no comprehensive evaluation of the preoperative diagnosis, such as an MRI or PET-CT assessment of the general condition at the first diagnosis. Due to the loss to follow-up after 8 years of treatment, the latest evaluation results of the treatment could not be reported, such as whether the treatment had a significant impact on the functional status and quality of life of the patient.

In conclusion, mediastinal EHE is quite rare in the clinic, and metastases and unresectability are associated with poor prognosis. Although no optimal treatment strategy has been established, curative surgery and appropriate adjuvant therapy, including radiotherapy and chemotherapy, are currently the main treatments. From the present case, we believe that the reasonable “take-away” lesson is that active treatment and prolonged surveillance can be essential for improving prognosis and prolonging survival.

## Data Availability Statement

The original contributions presented in the study are included in the article/supplementary material, further inquiries can be directed to the corresponding author/s.

## Ethics Statement

The study involving human participants was reviewed and approved by Institutional Review Board of Jinhua Hospital, Zhejiang University School of Medicine [Jinhua].Written informed consent was obtained from the patient for the publication of this case report.

## Author Contributions

JFP, QLZ, and BYC collected the patient's data. JFP, ZPM, and GSL analyzed the data and performed reference search. ZPM, QLZ, and GSL drafted and revised the manuscript. All authors contributed toward data analysis, drafting and revision of the manuscript, and read and approved the final manuscript.

## Conflict of Interest

The authors declare that the research was conducted in the absence of any commercial or financial relationships that could be construed as a potential conflict of interest.

## Publisher's Note

All claims expressed in this article are solely those of the authors and do not necessarily represent those of their affiliated organizations, or those of the publisher, the editors and the reviewers. Any product that may be evaluated in this article, or claim that may be made by its manufacturer, is not guaranteed or endorsed by the publisher.

## Abbreviations

EHE: Epithelioid haemangioendothelioma
IMRT: intensity modulated radiation therapy
CT: computed tomography
MRI: magnetic resonance imaging
T1WI: T1-weighted images
T2WI: T2-weighted images
PET-CT: positron emission tomography-computed tomography.
